# Benchmarking omics-based prediction of asthma development in children

**DOI:** 10.1186/s12931-023-02368-8

**Published:** 2023-02-26

**Authors:** Xu-Wen Wang, Tong Wang, Darius P. Schaub, Can Chen, Zheng Sun, Shanlin Ke, Julian Hecker, Anna Maaser-Hecker, Oana A. Zeleznik, Roman Zeleznik, Augusto A. Litonjua, Dawn L. DeMeo, Jessica Lasky-Su, Edwin K. Silverman, Yang-Yu Liu, Scott T. Weiss

**Affiliations:** 1 Channing Division of Network Medicine, Department of Medicine, Brigham and Women’s Hospital, Harvard Medical School, Boston, MA 02115 USA; 2 Department of Mathematics, University of Hamburg, 21109 Hamburg, Germany; 3 Genetics and Aging Research Unit, Department of Neurology, McCance Center for Brain Health, Mass General Institute for Neurodegenerative Disease, Massachusetts General Hospital, Harvard Medical School, Charlestown, MA USA; 4 Department of Radiation Oncology, Brigham and Women’s Hospital, Boston, MA USA; 5 Division of Pediatric Pulmonology, Golisano Children’s Hospital, Rochester, NY USA; 6 Center for Artificial Intelligence and Modeling, The Carl R. Woese Institute for Genomic Biology, University of Illinois at Urbana-Champaign, Urbana, IL 61801 USA

**Keywords:** Asthma, Disease status, Prediction, Multi-omics

## Abstract

**Background:**

Asthma is a heterogeneous disease with high morbidity. Advancement in high-throughput multi-omics approaches has enabled the collection of molecular assessments at different layers, providing a complementary perspective of complex diseases. Numerous computational methods have been developed for the omics-based patient classification or disease outcome prediction. Yet, a systematic benchmarking of those methods using various combinations of omics data for the prediction of asthma development is still lacking.

**Objective:**

We aimed to investigate the computational methods in disease status prediction using multi-omics data.

**Method:**

We systematically benchmarked 18 computational methods using all the 63 combinations of six omics data (GWAS, miRNA, mRNA, microbiome, metabolome, DNA methylation) collected in The Vitamin D Antenatal Asthma Reduction Trial (VDAART) cohort. We evaluated each method using standard performance metrics for each of the 63 omics combinations.

**Results:**

Our results indicate that overall Logistic Regression, Multi-Layer Perceptron, and MOGONET display superior performance, and the combination of transcriptional, genomic and microbiome data achieves the best prediction. Moreover, we find that including the clinical data can further improve the prediction performance for some but not all the omics combinations.

**Conclusions:**

Specific omics combinations can reach the optimal prediction of asthma development in children. And certain computational methods showed superior performance than other methods.

**Supplementary Information:**

The online version contains supplementary material available at 10.1186/s12931-023-02368-8.

## Background

Asthma is a chronic condition characterized by wheezing, coughing and reversible airflow obstruction [1]. The global prevalence, morbidity, mortality, and economic burden associated with asthma have been increasing in the past decades [2]. Advances in high-throughput sequencing technologies enable the availability of molecular assessments at the genome, epigenome, transcriptome, proteome, metabolome, and microbiome levels, providing the potential for a comprehensive understanding of human health and diseases [3–6]. Prediction of disease status, including asthma, is critical for understanding the etiology of the disease, discovering the molecular biomarkers and subsequentially identifying suitable interventions. Integrated approaches through combining multi-omics data from different biological layers might improve our ability to bridge the gap from genotype to phenotype [7–10].

Numerous computational methods have been developed to classify patients using their single- or multi-omics data. For example, ensemble-based methods, random forest, and gradient boost decision trees have shown superior performance over only using single-omics data or by directly concatenating the features from different omics data types for multi-omics classification tasks [11–13]. Moreover, several deep learning-based methods have been proposed for the classification in biomedical applications, generating higher performance than existing supervised multi-omics integration methods in various classification tasks [14, 15]. However, benchmarking those computational methods using various combinations of omics data for the disease status prediction has not been studied before. Note that for the disease status prediction, the omics data were collected before the disease onset, which is fundamentally different from the patient classification problem where the omics data were collected after the disease onset.

**Fig. 1:**
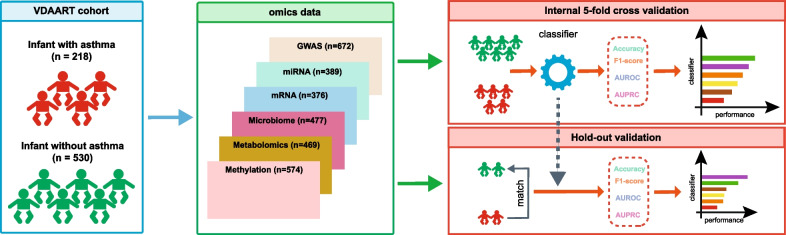
Workflow of the asthma development prediction. We collected six types of omics data taken at/before year 1 in the VDAART cohort: (1) GWAS: genome-wide SNP genotyping data and genome-wide association study analysis results; (2) Child miRNA (cord blood); (3) Child mRNA transcriptomics (cord blood); (4) Child microbiome 3–6 months; (5) Child metabolomics at 1 year; (6) Child DNA methylation data (cord blood). We split the subjects into two parts for hold-out validation and cross-validation, respectively. Then, we applied different classifiers to predict the asthma status at year 3 using the omics data. Each method was evaluated using Accuracy, F1 score, AUROC and AUPRC

Here, we compared different disease status prediction methods (using standard performance metrics) on six different types of omics data collected in *The Vitamin D Antenatal Asthma Reduction Trial* (VDAART) cohort [16]. Our aim is to identify the best prediction method and the best combination of omics data for the prediction of asthma development (see Fig. 1). Our results indicate that Logistic Regression, Multi-Layer Perceptron, and Graph Neural Network-based method MOGONET display superior performance and the combinations of transcriptional, genomic and microbiome data can yield the best prediction of asthma development. Moreover, we found that including the clinical covariates can further improve the prediction performance for some (but not all) omics combinations.

## Methods

### VDAART cohort

VDAART is a clinical trial to examine the hypothesis that vitamin D supplementation in pregnant women will prevent the development of asthma and allergies in their children [17, 18]. Pregnant women between 18 and 40 years of age and at an estimated gestational age between 10 and 18 weeks were recruited at three clinical centers: Boston Medical Center, Washington University at Saint Louis, and Kaiser Permanente Southern California Region. In the VDAART study, six types of omics data of the children have been collected: (1) GWAS: genome-wide SNP genotyping data and genome-wide association study analysis results. Genotyping of children in VDAART was performed on the Illumina Infinium HumanOmniExpressExome BeadChip, and SNP genotypes are called using the Illumina GenCall software. (2) child miRNA (cord blood); (3) child mRNA transcriptomics (cord blood). Total RNA was isolated from samples by the Qiagen miRNAeasy Serum/Plasma extraction kit and QIAcube automation. Small RNA sequencing libraries were prepared using the Norgen Biotek Small RNA Library Prep Kit and then sequenced on the Illumina NextSeq 500 platform at 51 bp single-end reads. (4) child microbiome at 3–6 months. DNA extractions were performed on stool samples, and the bacterial 16S rRNA gene (V3 to V5 hypervariable regions) was amplified. (5) child metabolomics at 1 year. Nontargeted global metabolomic profiles were generated at Metabolon Inc. by using ultra-performance liquid chromatography–tandem mass spectroscopy (UPLC-MS/MS). (6) child DNA methylation data (cord blood). Cord blood and peripheral blood DNA using the Qiagen Puregene Kit (Valencia, CA, USA) and bisulfite converted using the EZ DNA Methylation-Gold Kit (Zymo Research, Irvine, CA, USA). We randomized samples by chips and plates and generated DNA methylation data using the Infinium HumanMethylation450 BeadChip (Illumina, San Diego, CA, USA).

**Fig. 2:**
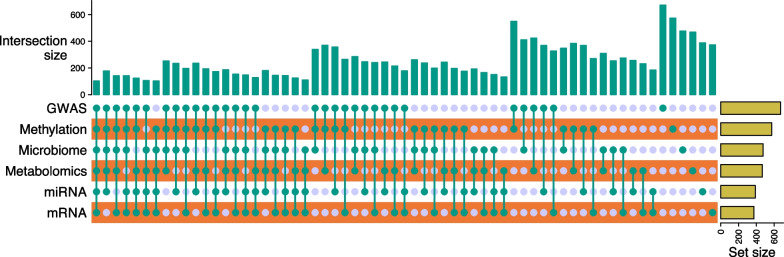
UpSet plot of the intersection of six types of omics data in the VDAART cohort. GWAS: genome-wide SNP genotyping data and genome-wide association study analysis results; (2) Child miRNA (cord blood); (3) Child mRNA transcriptomics (cord blood); (4) Child microbiome 3–6 months; (5) Child metabolomics at 1 year; (6) Child DNA methylation data (cord blood)

Among the 748 child participants in VDAART, 102 participants (13.6%) have all the six types of omics data available. Among the 6 omics data types, GWAS data has the largest sample size (see Fig. 2). Postnatally, every 3 months, questionnaires administered to the mother by telephone up to the child’s third birthday inquired about the health of the infant and child, especially the occurrence of wheezing illnesses and asthma and allergy symptoms and diagnoses. In-person visit for the child obtained yearly questionnaire data, determined anthropometric measurements, and collected blood. Here, we applied various machine learning models to predict the children’s asthma status at year 3 using those six omics data collected at/before year 1. Assessment of asthma was based on a doctor’s diagnosis which was defined as a positive response to a direct question to the mother at any time in the first three years of the life of the child. As recent symptoms may help identify young children with significant asthma [19], a more specific definition of doctor’s diagnosis plus symptoms and medication use in the past was used. In addition, the following were also collected in the VDAART study: vitamin D levels in blood of both the mother (through measurement of 25(OH)D levels in cord blood at delivery) and the child (at year 1); and other relevant covariates, e.g., maternal asthma, race and clinical center (see Table 1 for characteristic).

**Table 1:** Key Characteristics of VDAART subjects used in benchmarking asthma development prediction

Characteristics	Healthy (n = 249)	Asthmatic (n = 83)
Gender		
Male	128	33
Female	121	50
Race		
Asian	20	2
Black, African American	100	42
Native Hawaiian	2	1
White	86	30
Others	40	6
Mother’s age (year)	28.11 $\pm$ 5.66	27.15 $\pm$ 6.09
Mother’s gestation age in days, at enrollment	96.51 $\pm$ 18.78	101.11 + 19.76
Vitamin blood values (ng/ml) at Enrollment visit	24.42 $\pm$ 1046	23.00 $\pm$ 9.99
Site name		
Boston Medical Center	45	25
Kaiser Permanente Southern California Region	104	23
Washington University at Saint Louis	100	35

### Prediction methods and performance evaluation

We leveraged several classical classifiers in scikit-sklearn [20], i.e., k-Nearest Neighbors (KNN), Logistic Regression (LR), LRCV (Logistic Regression with cross-validator), Random Forest (RF), Multi-Layer Perceptron (MLP) and Gradient Boosting. We also considered two state-of-the-art deep learning methods: MOGONET [14] and Tabnet [21]. In addition, we also evaluated LR-VAE (Variational AutoEncoder) and LRCV-VAE, compressing the input dimension of miRNA, mRNA, microbiome, metabolomics and DNA methylation data to 5 via the variational autoencoder, which has been heavily used in dimension reduction for biological data [22, 23] (see Table 2 for the list of prediction methods). To compare the performance of different methods on prediction of asthma status, we first split the subjects into two groups for the following evaluation purposes: (1) Hold-out validation: among the 102 subjects that have all six omics data types available, we randomly chose 16 cases, then randomly selected 16 controls whose race and clinical center match each case. (2) Cross-validation: fivefold cross-validation was used to evaluate the performance of each classification method on the remaining subjects (in total 300). To evaluate the performance of each method, we used the standard classification performance metrics: (1) Accuracy; (2) F1-score; (3) AUROC: Area Under the Receiver Operating Characteristic (ROC) curve and (4) AUPRC: Area Under the Precision-Recall Curve (PRC).

**Table 2:** Prediction models for asthma development

Method	Description	Refs.
Linear models		
LR	Logistic Regression models the probability of object belonging to a class by having the log-odds for the class to be a linear combination of features	[42]
LRCV	Logistic Regression with build-in validation support to find the optimal parameters	[42]
LR-VAE	Logistic Regression with reduced features using VAE (Variational AutoEncoder)	[43, 44]
LRCV-VAE	LRCV-VAE: Logistic Regression with build-in validation support to find the optimal parameters and reduced features using VAE	[43, 44]
Nearest neighbors		
KNN	*k*-nearest neighbors algorithm that predicts the class of object to the class of most common among its *k* nearest neighbors	[45]
Support vector machine		
SVC	*C*-Support Vector Classification is a method for classification by constructing a set of hyperplanes in high dimensional space	[46]
Ensemble methods		
AdaBoost	AdaBoost algorithm is an iterative procedure that tries to approximate the Bayes classifiers by combining many weak classifiers	[47, 48]
GTB	Learning procedure in Gradient Tree Boosting consecutively fit new models to provide a more accurate estimate of the response variable	[49, 50]
RF	Random forest is an ensemble classifier by constructing many decision trees and the final prediction is selected by most trees	[51]
Bagging	Bagging algorithm is a method for generating multiple versions of a predictor, then using these predictions to get an aggregated predictor	[52]
Ensemble	Aggregate the predictions of all other classifiers together. The continuous probability of a subject being asthmatic is the average probabilities of 15 methods, and a subject is predicted as asthmatic if it was predicted as asthmatic by at least 7 methods	
Decision trees		
DecisionTree	Decision Trees predict the response value by learning simple decision rules inferred from the data features	[53]
ERT	An extremely randomized tree classifier is a tree-based ensemble method consisting of randomizing strongly both attribute and cut point choice	[54]
Naïve Bayes		
BernoulliNB	Implements the Naïve Bayes training and classification for data that is distributed based on multivariate Bernoulli distribution	[55]
GaussianNB	Implements the Naïve Bayes training and classification for data that is distributed based on multivariate Gaussian distribution	[56]
Neural networks		
MLP	Multi-layer Perceptron in a fully connected feedforward neural networks with at least three layers	[57]
MOGONET	MOGONET is a multi-omics data analysis framework for classification tasks utilizing graph convolutional networks	[14]
Tabnet	Tabnet uses a canonical deep neural networks architecture for tabular data with interpretability	[21]

### Feature selection

Omics data is typically high-dimensional in the sense that the number of features is significantly larger than the number of samples [24, 25]. Feature selection can filter out irrelevant and redundant features by identifying a subset of relevant features [26]. Besides, when fewer features are used as inputs in machine learning models, it also minimizes over-fitting risks. Numerous methods can be used for feature selection, e.g., univariate statistical testing, feature variance, Random Forest importance ranking, and information-theoretic measures [15, 27]. Here, we used the Wilcoxon rank-sum test on cross-validation subjects to identify the key features of count data, including miRNA, mRNA and microbiome data, due to its solid False Discover Rate (FDR) control and good power [28] (see Additional file 1: sec.2 for detail of statistical analysis). For each of those data types, the top 300 features with the lowest p-values were selected, so that the number of features is comparable to the number of subjects (249 healthy controls and 83 asthmatic cases). For continuous metabolomics and methylation data, we used the feature variance to identify the top 300 features with the largest variance across subjects [29]. We reduced the genetic data to 4 polygenic scores (PGS) computed from previous work [30] and 2 SNPs (rs4795399 and rs117097909) in the established 17q21 locus [31].

### Omics data imputation

Since not all six omics data types are available for each subject, we performed data imputation first so that the evaluation of each prediction method was performed on the same set of subjects, enabling us to systematically examine the capability of each omics in the prediction of asthma development. To keep more omics data unimputed and the subject size maximized, we selected the subjects with the following three omics data types: GWAS, DNA methylation and the microbiome all available. Then, we imputed the miRNA, mRNA and metabolomics data using the following three methods, respectively: (1) median imputation: the missing value of a feature is replaced with the median value of the other samples. (2) TOBMI [32] (trans-omics block missing data): missing data of a subject in one omics is the weighted combination of k-nearest neighbors identified from another omics data. Here, the missing values of miRNA and mRNA were imputed using a k-nearest neighbors (KNN) weighted method, where a gene expression of a missing subject is the weighted combination of *k* nearest neighbors identified using the DNA methylation data. We leveraged this idea to impute the metabolomics data using the microbiome data. Hence, the distance matrix was constructed from the microbiome data. (3) missForest [33]: an iterative imputation method based on a random forest classifier. 66% subjects were missing one omics data type, 28% subjects were missing two omics data types, and only 5% subjects were missing all three omics data types. Note that, imputing the missing data on the original omics data requires significantly high computational effort, so we performed the imputation process after the feature selection. We emphasize that the imputation here is subject based, in the sense that the entire omics of some subjects were missing, rather than only few features within an omics were missing. Therefore, some traditional imputation methods, i.e., k-nearest neighbors cannot be directly utilized.

## Results

### Heathy and asthmatic children show differences in their multi-omics profiles

**Fig. 3:**
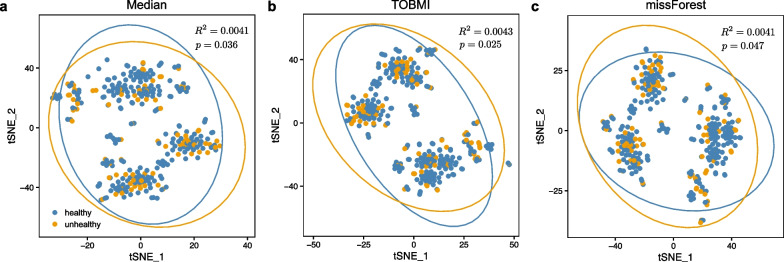
Distribution of healthy controls and asthmatic cases. TSNE (t-distributed stochastic neighbor embedding) plot shows that healthy (blue; $n=249$) and nonhealthy (orange; $n=83$) groups have significantly different distributions of omics profiles according to PERMANOVA (Euclidean distance by directly concatenating the features from six types of omics. The feature number of GWAS is 6 and 300 for the each of remaining five omics.). Each point corresponds to a subject. Ellipses correspond to 95% confidence regions

We firstly examined the differences in the imputed multi-omics profiles between the healthy controls ($n=249$) and asthmatic cases ($n=89$). We found a significant difference between the distributions of healthy and asthmatic groups using the t-SNE visualization (permutational multivariate analysis of variance (PERMANOVA), $P<0.05$), regardless of imputation methods (see Fig. 3).

### There are four consistently high-performing methods in the cross-validations

**Fig. 4:**
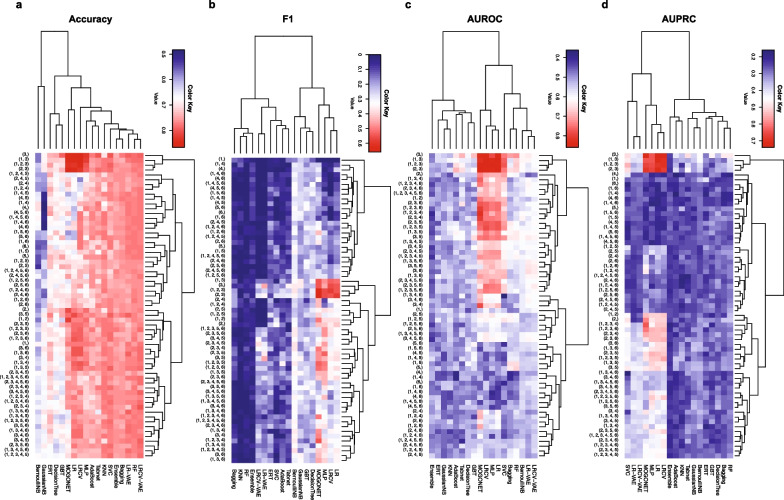
Prediction performance of prediction methods using all six omics combinations in cross-validation. Each classifier is applied to predict the asthma status of children at year 3 using all six omics combinations (in total 63). The missing values of miRNA, mRNA and metabolomics data were imputed using the median values. The heatmap plot shows the average performance of fivefold cross-validations. AdaBoost: A decision-theoretic generalization of online learning and an application to boosting [47]; Bagging: ensemble meta-estimators that aggregate individual predictions to a final prediction [52]; BernoulliNB: Bernoulli Naïve Bayes [55]; GTB: Gradient Tree Boosting [49]; DecisionTree: Decision Trees [53]. Ensemble: aggregate the prediction of all other classifiers together. ERT: An extremely randomized tree classifier [54]. GaussianNB: Gaussian Naïve Bayes. KNN: k-nearest neighbors; LR: Logistic Regression; LRCV: Logistic Regression with build-in validation support to find the optimal parameters; LR-VAE: Logistic Regression with compressed features from VAE (Variational AutoEncoder); LRCV-VAE: Logistic Regression with build-in validation support to find the optimal parameters and compressed feature from VAE (Variational AutoEncoder); MLP: Multi-layer Perceptron; MOGONET: Multi-Omics Graph Convolutional Networks [14]; RF: Random Forest; SVC: Support Vector Classification; Tabnet: Attentive Interpretable Tabular Learning [21]. 1: GWAS; 2: miRNA; 3: mRNA; 4: Microbiome; 5: Metabolomics; 6: DNA methylation

Among all tested methods in fivefold cross-validations, we found that LR, LRCV, MLP and MOGONET show relatively higher performance over all four types of evaluation metrics (imputed using the median). For example, the highest Accuracy, F1, AUROC and AUPRC of LRCV are 0.92, 0.8, 0.96 and 0.89 among fivefold cross-validations. MOGONET is a novel multi-omics integrative method that jointly explores omics-specific learning and cross-omics correlation learning based on Graph Convolutional Networks (GCN) showing similar performance to LRCV (see Fig. 4; Additional file 1: Fig. S1). In particular, we found that the performance of those top-ranking methods is robust to different imputation methods (see Additional file 1: Fig. S2, S3 for missForest and TOBMI imputation). Higher performance of those four methods implies that prediction of children’s asthma development through leveraging the rich information in multi-omics is feasible.

### Transcriptional and genomic data are critical for asthma prediction

Figure 4 shows the predictive performance of each prediction method across all possible combinations of six omics data types. We observed that the prediction performance largely depends on the omics used. To examine the importance of different omics combinations on children’s asthma status prediction, we ranked those 63 combinations from six omics data types based on their median performance across all prediction methods. Interestingly, we found a consistent omics importance ranking over four evaluation metrics: mRNA alone, and combinations of GWAS, miRNA and mRNA can achieve the highest performance. Especially, mRNA alone shows the highest ranking among Accuracy, AUROC and AUPRC (see Additional file 1: Fig. S4). Furthermore, we measured the importance of each feature (such as gene, mRNA, miRNA) using MOGONET, since it yields the overall best performance with omics combination of genome, miRNA, and mRNA data yields the overall best performance, we selected this omics combination and the feature importance in MOGONET was computed by the performance decrease, e.g., F1 score after the feature is removed. We found biomarkers (i.e., features with high importance scores) identified by MOGONET have also shown associations with asthma (see Additional file 1: Table S1). For example, has-miR-581, a microRNA downregulated in severe asthma, is associates with forced expiratory volume in 1 s (FEV1) and immune inflammation [34]. In addition, hsa-miR-376c-3p, hsa-miR-374b-5p, hsa-miR-374c-5p et al., are circulating microRNAs associated with lung function in asthma [35]. When compared to healthy controls, bronchial smooth muscle cells from asthmatic patients express different levels of hsa-miR-376a-3p and hsa-miR-330-5p [36]. ENSG00000267174 is a long noncoding RNA (lncRNA), and many lncRNAs have been shown to be associated with asthma severity or inflammatory phenotype [37]. ENSG00000004139 can regulate the cell survival and cytokine release after inflammasome activation [38]. Again, we found that those top-ranking omics combinations are quite robust to different imputation methods. These results suggest that accurate prediction of asthma development in children does not require sequencing as many as possible omics data. Whereas, using transcriptional with genomic data can yield superior performance for predicting asthma development at year 3.

### Different imputation methods produce a similar performance

Although multi-omics analysis can provide the connections between biomolecules from different layers of omics data, one of the key challenges in multi-omics approaches is missing values within and across the omics data. Missing values across omics are a particular concern as they will result in different sample sizes among the omics, which requires imputation for the downstream analyses, i.e., classification. We compared the prediction performance of each prediction method using all 63 omics combinations imputed with three different methods, showing that median and TOBMI imputations can achieve significantly higher AUPRC than missForest (see Additional file 1: Fig. S5). Yet, the overall performance of the three imputation methods is similar.

### Hold-out validation displays similar results to cross-validations

**Fig. 5:**
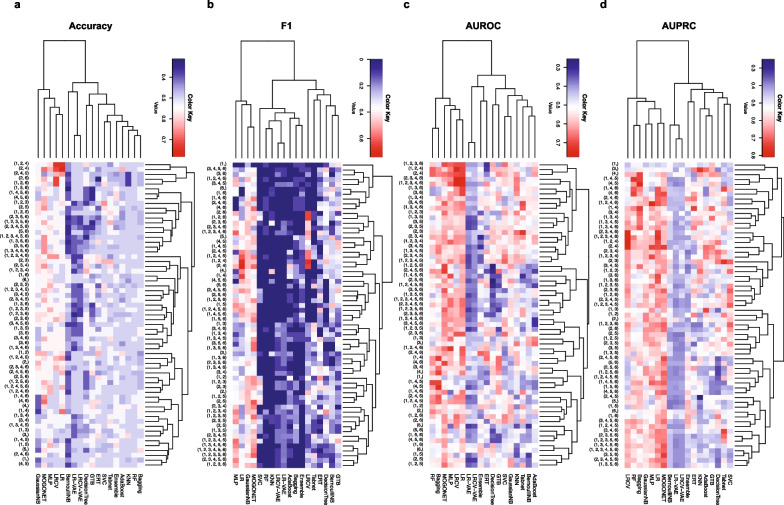
Prediction performance of prediction models using all six omics combinations for hold-out set validation. Each classifier is applied to predict the asthma status of children at year 3 using all six omics combinations (in total 63). The missing values of miRNA, mRNA and metabolomics data were imputed using the median values. The heatmap plot shows the performance of each prediction method in 32 balanced subjects with 16 healthy controls and 16 asthmatic cases. 1: GWAS; 2: miRNA; 3: mRNA; 4: Microbiome; 5: Metabolomics; 6: DNA methylation

Phenotypes in biological studies are typically imbalanced; for example, most binary traits have fewer cases than controls [39]. To examine the performance of each prediction method on a balanced data set without imputation, we trained each method using all the 300 subjects in fivefold cross-validations, then evaluated them using an additional 32 subjects with 16 healthy controls and 16 asthmatic cases, respectively. Again, we found that LR and MOGONET show superior performance over other methods, i.e., the Accuracy, F1, AUROC and AUPRC of LR were 0.78, 0.74, 0.70 and 0.72, respectively, and 0.69, 0.59, 0.66 and 0.75 for MOGONET (see Fig. 5; Additional file 1: Fig. S6). In addition, we found that the combination of miRNA and mRNA achieves the highest Accuracy and AUPRC. Yet, the combination of miRNA and microbiome data can produce the highest F1 and AUROC (see Additional file 1: Fig. S7).

### Utilizing covariates can further improve the prediction performance for particular omics combination

**Fig. 6:**
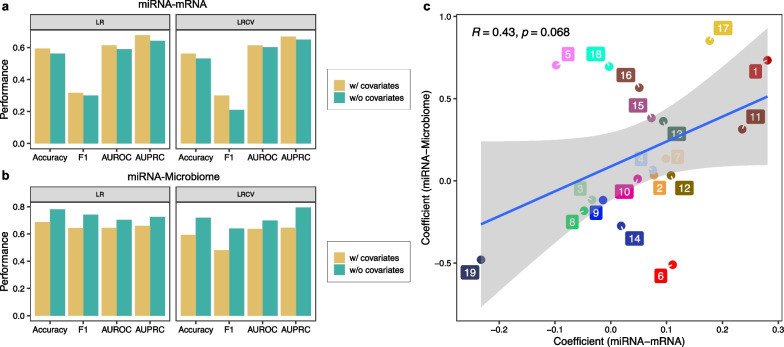
Comparison of prediction performance with and without using covariates. The performance of two classifiers: LR and LRCV were compared in predicting the asthma outcome of children at year 3 before and after using covariates together with using the miRNA and mRNA (**a**) and miRNA and microbiome (**b**), respectively. The missing values of miRNA, mRNA data were imputed using the median values. w/covariates: including covariates in prediction; w/o covariates: without using covariates in prediction. **(c):** Correlation between the coefficient of each covariate together with two omics combinations: (1) miRNA and mRNA and (2) miRNA and microbiome in the prediction of asthma status using Logistic Regression. 1: History of asthma in mother; 2: History of asthma in father. 3: History of hay fever or allergic rhinitis in mother; 4: History of eczema or atopic dermatitis in mother; 5: History of eczema or atopic dermatitis in father; 6: History of hay fever or allergic rhinitis in mother; 7: Marital status of mother; 8: Highest level of school mother has completed; 9: Ethnicity of mother; 10: Race of mother; 11: Enrollment site; 12: assignment; 13: Treatment Arm; 14: Vitamin D blood value (ng/ml) at Enrollment visit; 15: Mother's age at enrollment; 16: Mother's gestation age in days, at enrollment; 17: Mother's Education re-worked; 18: Mother's Marital status; 19: Race/Ethnicity

To evaluate whether including covariates together with omics data can further improve the prediction performance, we considered the following covariates associated with each subject, i.e., father and mother’s asthma status, race, as well as vitamin D level into the prediction model. Previous analysis in hold-out validation using all 63 omics combinations has shown that the combination of miRNA and mRNA or the combination between miRNA and microbiome omics can reach the optimal performance for most of the prediction methods. Here we intended to investigate the influence of covariates by examining the performance of each method before and after including those covariates in addition to best-performing omics combinations. As those covariates cannot be included easily in all prediction models, i.e., treating these covariates as an additional omics data type for MOGONET, we focused on two promising methods LR and LRCV that can fully exploit all predictors fairly. We found that that the impact of covariates on the asthma prediction depends on the omics used, e.g., it can further improve the prediction for miRNA and mRNA combination for both of LR and LRCV, regardless of the performance metrics (see Fig. 6a). Yet, including those covariates will decrease the prediction performance for the miRNA and microbiome combination (see Fig. 6b). To understand this difference, we examined the association between coefficients of each covariate in LR using two omics combinations, respectively, finding that the coefficients from two omics combinations display a positive correlation. Yet, we do find that for some covariates, such as, history of eczema or atopic dermatitis in mother, mother’s marriage status and history of hay fever or allergic rhinitis in mother are associated with high coefficients in one combination, but not for another.

## Discussion

The global prevalence, morbidity, mortality and economic burden of children’s asthma has significantly increased in the past 40 years [1]. Predicting asthma development for children is imperative to understand the etiology of the disease and identify suitable interventions [10]. Yet, many diseases (including asthma) are heterogeneous, which renders the prediction of the disease status a big challenge. Here, we leveraged the rich omics collected in the VDAART cohort, examining the existing classification methods in the prediction of children’s asthma development at year 3 using multi-omics data collected at/before year 1. Our results imply that including a subset of all types of omics data is helpful in asthma outcome prediction, especially a combination of transcriptional, genomic and microbiome data can achieve optimal prediction. In addition, the imputation methods for missing values do not show a significant impact on the prediction.

Our analysis related to the impact of covariates on the asthma development prediction suggests that including the covariates in the prediction models does not always improve the performance. This also implies that the conclusion drawn from VDAART can also be valid in other cohorts, i.e., compromised of subjects with different racial distribution, as, in this study, race is not an importance predictor. However, we acknowledge the importance of replicating these findings in additional diverse populations.

Vitamin D can impact the developing of the lung and immune system during the fetal and early postnatal periods [40, 41], thus deficiency of vitamin D in pregnancy may be important in early asthma and wheezing. The VDAART Randomized Clinical Trial implies that the 3-year incidence of asthma or recurrent wheeze in the infants was 24.3% with 4400-IU/d and 30.4% with a 400-IU/d supplement [17]. This reduction demonstrates that supplementation of vitamin D may be an important intervention for child health. The prediction of children’s asthma development after including the covariates indicates that vitamin D level is associated with a reduction (negative coefficient) in the relative risk of asthma if the prediction is accurate, for instance, using the combination of miRNA and microbiome omics data types. This confirms that supplementation of vitamin D in pregnancy can reduce the risk of asthma for children.

Omics data usually contains missing values. Integration of those omics data together typically requires all omics of each subject available, which is challenging as more types of omics data are included. Data imputation enables us to systematically examine the impact of each omics data type in the prediction of disease status. Our results demonstrate that the performance of those superior methods, i.e., Logistic Regression using combinations of non-imputed omics, i.e., miRNA and microbiome still displayed superior performance than other methods.

## Supplementary Information

**Additional** **file** **1:** **Figure** **S1:** Prediction performance of each prediction method in cross-validation. **Figure** **S2**: Prediction performance of classification models using all six omics combinations in cross-validation imputed by *TOMBI*. **Figure** **S3**: Prediction performance of classification models using all six omics combinations in cross-validation imputed by *missForest*. **Figure** **S4:** Omics combination importance in cross-validation. **Table S1**: Important omics biomarkers identified by MOGONET using genome, miRNA and mRNA data. Fig**ure** **S5:** Performance comparison between different imputation methods. **Figure** **S6:** Omics combination importance in hold-out validation. **Figure** **S7:** Prediction performance of each method in hold-out validation.

## Data Availability

The data presented in this study are available upon request.

## References

[1] Braman SS (2006). The global burden of asthma. Chest.

[2] Caffrey Osvald E, Bower H, Lundholm C (2020). Asthma and all-cause mortality in children and young adults: a population-based study. Thorax.

[3] Di Resta C, Galbiati S, Carrera P (2018). Next-generation sequencing approach for the diagnosis of human diseases: open challenges and new opportunities. Ejifcc.

[4] Grada A, Weinbrecht K (2013). Next-generation sequencing: methodology and application. J Invest Dermatol.

[5] Kilpinen H, Barrett JC (2013). How next-generation sequencing is transforming complex disease genetics. Trends Genet.

[6] Ku CS, Naidoo N, Wu M (2011). Studying the epigenome using next generation sequencing. J Med Genet.

[7] Bersanelli M, Mosca E, Remondini D (2016). Methods for the integration of multi-omics data: mathematical aspects. BMC Bioinformatics.

[8] Graw S, Chappell K, Washam CL (2021). Multi-omics data integration considerations and study design for biological systems and disease. Mol Omics.

[9] Hasin Y, Seldin M, Lusis A (2017). Multi-omics approaches to disease. Genome Biol.

[10] Subramanian I, Verma S, Kumar S (2020). Multi-omics data integration, interpretation, and its application. Bioinform Biol Insights.

[11] Picard M, Scott-Boyer M-P, Bodein A (2021). Integration strategies of multi-omics data for machine learning analysis. Comput Struct Biotechnol J.

[12] Xie G, Dong C, Kong Y (2019). Group lasso regularized deep learning for cancer prognosis from multi-omics and clinical features. Genes.

[13] Chaudhary K, Poirion OB, Lu L (2018). Deep learning-based multi-omics integration robustly predicts survival in liver cancerusing deep learning to predict liver cancer prognosis. Clin Cancer Res.

[14] Wang T, Shao W, Huang Z (2021). MOGONET integrates multi-omics data using graph convolutional networks allowing patient classification and biomarker identification. Nat Commun.

[15] Rohart F, Gautier B, Singh A (2017). mixOmics: an R package for ‘omics feature selection and multiple data integration. PLoS Comput Biol.

[16] Group CAMPR. The childhood asthma management program (CAMP): design, rationale, and methods. Controlled clinical trials 1999; 20:91–120. 10027502

[17] Litonjua AA, Carey VJ, Laranjo N (2016). Effect of prenatal supplementation with vitamin D on asthma or recurrent wheezing in offspring by age 3 years: the VDAART randomized clinical trial. JAMA.

[18] Weiss ST, Litonjua AA (2016). Can we prevent childhood asthma before birth? Summary of the VDAART results so far. Expert Rev Respir Med.

[19] Galant SP, Morphew T, Amaro S (2006). Current asthma guidelines may not identify young children who have experienced significant morbidity. Pediatrics.

[20] Buitinck L, Louppe G, Blondel M, et al. API design for machine learning software: experiences from the scikit-learn project. arXiv preprint arXiv:1309.0238 2013.

[21] Arik SO, Pfister T. TabNet: Attentive Interpretable Tabular Learning. arXiv:1908.07442 [cs, stat] 2020.

[22] Lin E, Mukherjee S, Kannan S (2020). A deep adversarial variational autoencoder model for dimensionality reduction in single-cell RNA sequencing analysis. BMC Bioinformatics.

[23] Wang D, Gu J (2018). VASC: dimension reduction and visualization of single-cell RNA-seq data by deep variational autoencoder. Genomics Proteomics Bioinformatics.

[24] Leclercq M, Vittrant B, Martin-Magniette ML (2019). Large-scale automatic feature selection for biomarker discovery in high-dimensional OMICs data. Front Genet.

[25] Moon KR, van Dijk D, Wang Z (2019). Visualizing structure and transitions in high-dimensional biological data. Nat Biotechnol.

[26] Bommert A, Sun X, Bischl B (2020). Benchmark for filter methods for feature selection in high-dimensional classification data. Comput Stat Data Anal.

[27] Du W, Cao Z, Song T (2017). A feature selection method based on multiple kernel learning with expression profiles of different types. BioData Mining.

[28] Li Y, Ge X, Peng F (2022). Exaggerated false positives by popular differential expression methods when analyzing human population samples. Genome Biol.

[29] Zhuang J, Widschwendter M, Teschendorff AE (2012). A comparison of feature selection and classification methods in DNA methylation studies using the illumina infinium platform. BMC Bioinformatics.

[30] Sordillo JE, Lutz SM, Jorgenson E (2021). A polygenic risk score for asthma in a large racially diverse population. Clin Exp Allergy.

[31] Ferreira MA, Mathur R, Vonk JM (2019). Genetic architectures of childhood-and adult-onset asthma are partly distinct. Am J Hum Genet.

[32] Dong X, Lin L, Zhang R (2019). TOBMI: trans-omics block missing data imputation using a k-nearest neighbor weighted approach. Bioinformatics.

[33] Stekhoven DJ, Buhlmann P (2012). MissForest-non-parametric missing value imputation for mixed-type data. Bioinformatics.

[34] Francisco-Garcia AS, Garrido-Martín EM, Rupani H (2019). Small RNA species and microRNA profiles are altered in severe asthma nanovesicles from broncho alveolar lavage and associate with impaired lung function and inflammation. Noncoding RNA.

[35] Kho AT, Sharma S, Davis JS (2016). Circulating MicroRNAs: association with lung function in asthma. PLoS ONE.

[36] Alexandrova E, Miglino N, Hashim A (2016). Small RNA profiling reveals deregulated phosphatase and tensin homolog (PTEN)/phosphoinositide 3-kinase (PI3K)/Akt pathway in bronchial smooth muscle cells from asthmatic patients. J Allergy Clin Immunol.

[37] Gysens F, Mestdagh P, de Bony de Lavergne E (2022). Unlocking the secrets of long non-coding RNAs in asthma. Thorax.

[38] Carty M, Kearney J, Shanahan KA (2019). Cell survival and cytokine release after inflammasome activation is regulated by the Toll-IL-1R protein SARM. Immunity.

[39] Zhou W, Nielsen JB, Fritsche LG (2018). Efficiently controlling for case-control imbalance and sample relatedness in large-scale genetic association studies. Nat Genet.

[40] Zosky GR, Berry LJ, Elliot JG (2011). Vitamin D deficiency causes deficits in lung function and alters lung structure. Am J Respir Crit Care Med.

[41] Yurt M, Liu J, Sakurai R (2014). Vitamin D supplementation blocks pulmonary structural and functional changes in a rat model of perinatal vitamin D deficiency. Am J Physiol Lung Cell Mol Physiol.

[42] Tolles J, Meurer WJ (2016). Logistic regression: relating patient characteristics to outcomes. JAMA.

[43] Doersch C. Tutorial on Variational Autoencoders. arXiv:1606.05908 [cs, stat] 2016.

[44] Arnold TB (2017). kerasR: R Interface to the keras deep learning library. J Open Source Softw.

[45] Altman NS (1992). An introduction to kernel and nearest-neighbor nonparametric regression. Am Stat.

[46] Chang C-C, Lin C-J (2011). LIBSVM: a library for support vector machines. ACM Trans Intell Syst Technol.

[47] Freund Y, Schapire RE (1997). A decision-theoretic generalization of on-line learning and an application to boosting. J Comput Syst Sci.

[48] Hastie T, Rosset S, Zhu J (2009). Multi-class AdaBoost. Statis Interface.

[49] Friedman JH (2001). Greedy function approximation: a gradient boosting machine. Annal Statis.

[50] Natekin A, Knoll A (2013). Gradient boosting machines, a tutorial. Front Neurorobot.

[51] Ho TK. Random decision forests. In: Proceedings of 3rd international conference on document analysis and recognition 1995; 1:278–282

[52] Breiman L (1996). Bagging predictors. Mach Learn.

[53] Loh W-Y (2011). Classification and regression trees. Wiley Interdiscip Rev Data Mining Knowl Discov.

[54] Geurts P, Ernst D, Wehenkel L (2006). Extremely randomized trees. Mach Learn.

[55] McCallum A, Nigam K. A comparison of event models for naive bayes text classification. In: AAAI-98 workshop on learning for text categorization 1998; 752:41–48.

[56] Zhang H (2004). The optimality of naive Bayes. Aa.

[57] Hinton GE. Connectionist learning procedures. Mach Learn. 1990; 555–610.

