# Supplementary material for: Benchmarking omics-based prediction of asthma development in children
Source: Respir Res. 2023 Feb 26;24:63. doi: 10.1186/s12931-023-02368-8 (PMC9969629; doi:10.1186/s12931-023-02368-8)
Supplement: Supplementary file 1 — Additional file 1: Figure S1: Prediction performance of each prediction method in cross-validation. Figure S2: Prediction performance of classification models using all six omics combinations in cross-validation imputed by TOMBI. Figure S3: Prediction performance of classification models using all six omics combinations in cross-validation imputed by missForest. Figure S4: Omics combination importance in cross-validation. Table S1: Important omics biomarkers identified by MOGONET using genome, miRNA and mRNA data. Figure S5: Performance comparison between different imputation methods. Figure S6: Omics combination importance in hold-out validation. Figure S7: Prediction performance of each method in hold-out validation. [file 12931_2023_2368_MOESM1_ESM.docx]

**Benchmarking omics-based prediction of asthma development in children**

**Additional file 1**

[1. Benchmarking Prediction Methods 2](#_Toc109976173)

[1.1 Linear Models 2](#_Toc109976174)

[1.2 Nearest Neighbors 2](#_Toc109976175)

[1.3 Support Vector Machine. 2](#_Toc109976176)

[1.4 Ensemble methods 3](#_Toc109976177)

[1. 5 Decision Trees 4](#_Toc109976178)

[1.6 Naïve Bayes 4](#_Toc109976179)

[1.7 Neural Networks 4](#_Toc109976180)

[2. Statistical Analysis 5](#_Toc109976181)

# 1. Benchmarking Prediction Methods

## 1.1 Linear Models

**Logistic regression (LR).** Logistic regression^37^ is a classifier using a logistic function to model a binary output variable. According to the penalty term, logistic regression minimizes the following case function.

(1) L2 penalized logistic regression.

$L=\min_{w,c} \frac{1}{2}\boldsymbol{w}^{T}\boldsymbol{w}+C\sum_{i=1}^{N} log(\exp\left( -y_{i}\left( X_{i}^{T}\boldsymbol{w}+c \right) \right)+1)$.

(2) L1 penalized logistic regression.

$L=\min_{w,c} {\parallel\boldsymbol{w}\parallel}_{1}+C\sum_{i=1}^{N} log(\exp\left( -y_{i}\left( X_{i}^{T}\boldsymbol{w}+c \right) \right)+1)$.

(3) Elastic-Net regulation that combines the L1 and L2.

$L=\min_{w,c} \frac{1-\rho}{2}\boldsymbol{w}^{T}\boldsymbol{w}+\rho\parallel w\parallel+C\sum_{i=1}^{N} log(\exp\left( -y_{i}\left( X_{i}^{T}\boldsymbol{w}+c \right) \right)+1)$.

Here $\rho$ is used to control the contribution of L1 and L2 regulations. $X$ is the feature, $y$ is the outcome and $\boldsymbol{w}$ is the weight. In our calculation, we used L2 penalized logistic regression. Parameters of Linear regression were searched using grid to maximize AUROC as follows. ‘penalty’: ‘l2’; ‘C’: [100, 10, 1, 0.1, 0.01]; ‘solver’: ['lbfgs’, ‘newton-cg’, ‘liblinear’].

**Logistic regression CV (LRCV).** Logistic regression^37^ CV is another implementation of Logistic regression that can find the optimal parameters using the built-in cross-validation. Parameters of Linear regression CV were searched using grid to maximize AUROC as follows. ‘penalty’: ‘l2’; ‘solver’: ['lbfgs’, ‘newton-cg’, ‘liblinear’].

**Logistic regression (Logistic regression CV) with dimension reduction of Variational AutoEncoder**^38,39^ **(LR-VAE, LRCV-VAE).** We used the Variational AutoEncoder (VAE) to reduce the original dimension (300) of each omics types: miRNA, mRNA, Microbiome, Metabolomics and DNA methylation to 5. Then, those reduced features were fed into LR and LRCV to predict asthma status.

## 1.2 Nearest Neighbors

**K-nearest neighbors (KNN).** K-nearest neighbors^40^ is a non-parametric supervised learning classifier. This classifier first identifies the k nearest instances in the training set for each test instance based on Euclidean distance. Then, the predicted label of the new instance is assigned as the label of most neighbors having. Parameters of k-nearest neighbors were searched using grid to maximize AUROC as follows. ‘leaf_size’: ‘[1, 2, 5, 10, 50]’; ‘n_neighbors’: [1, 2, 5, 10, 50]; ‘p: [1, 2, 3, 4, 5].

## 1.3 Support Vector Machine.

**Support Vector Machine (SVM).** Support Vector Machine^41^ is a supervised algorithm that can be used to solve both classification and regression problems. Here, we used *C*-support vector classification (*C*-SVC) implementation in scikit-sklearn^30^. *C*-SVC solves the following primal optimization problem^53^.

$$\min_{w,b,\xi} \frac{1}{2}\boldsymbol{w}^{T}\boldsymbol{w}+C\sum_{i=1}^{N} \xi_{i}$$

subject to $y_{i}\left( \boldsymbol{w}^{T}\phi\left( X_{i} \right)+b \right)\geq1-\xi_{i}$, $\xi_{i}\geq0$

where $\phi\left( X_{i} \right)$ maps $X_{i}$ into a higher-dimensional space and $C>0$ is the regulation parameter. Parameters of Support Vector Machine were searched using grid to maximize AUROC as follows. ‘C’: [0.1, 1, 10, 100]; ‘gamma’: [0.001, 0.01, 0.1, 1]; ‘kernel’: ['rbf’, ‘poly’, ‘sigmoid’].

## 1.4 Ensemble methods

**Adaptive Boosting (AdaBoost).** AdaBoost^42,43^ is to find a final hypothesis with low error relative to a given distribution $D$ usually be set to be uniform, i.e., $D\left( i \right)=1/N$ over the training samples. The weights in AdaBoost at iteration $t$ is represented as $\boldsymbol{w}^{t}$ over the training samples. A distribution $\boldsymbol{p}^{t}$ is calculated by normalizing the weights $\boldsymbol{w}^{t}$ on each iteration $t$. This distribution is fed to the weak learner which generates a hypothesis $h_{t}$ that has small error with respect to the distribution. The boosting algorithm will generate the new weight vector $\boldsymbol{w}^{t+1}$ using the new hypothesis $h_{t}$. After several iterations $T$, the final hypotheisis is obtained which combines the outputs of the $T$ weak hypotheses using a weighted majority vote. Parameters of AdaBoost were searched using grid to maximize AUROC as follows. ‘learning_rate’: [0.001, 0.005, 0.01, 0.05, 0.1, 0.15]; ‘n_estimators’: [200, 500, 1,000, 2,000].

**Gradient Tree Boosting (GTB).** Gradient boosting machines^44,45^ (GBMs) are powerful machine learning methods that can learn with respect to different loss functions. The key learning procedure in GBMs is to fit consecutively new models to provide a more accurate estimate of the response variable^45^. This algorithm constructs the new base-learners to be maximally correlated with the negative gradient of the loss function^45^. Gradient Tree Boosting is one of GBM which uses the decision trees as the base-learner models. Parameters of AdaBoost were searched using grid to maximize AUROC as follows. ‘learning_rate’: [0.001, 0.005, 0.01, 0.05, 0.1, 0.15]; ‘n_estimators’: [200, 500, 1,000, 2,000]; ‘max_depth’: [10, 20, 50, 100, 500, None].

**Random Forest (RF).** RF^46^ uses decision trees as the base learner. Each tree is a non-linear model constructed with many linear boundaries. Each node in a decision tree is associated with a question asking about the data based on the value of a particular feature. The node in the present layer will be split into two children nodes in the next layer according to the question. A decision tree can be built by repeating this splitting in a greedy and recursive procedure until maximum depth has been achieved. During the splitting (i.e., the training process), RF randomly selects bootstrapped samples from the original data and a randomly selected subsets of features is used to evaluate the model. Finally, RF combines many decision trees into a single ensemble model and makes prediction by aggregating the prediction of all the individual trees. Parameters of RF were searched using grid to maximize AUROC as follows. ‘boostrap: [True, False]; ‘n_estimators’: [200, 500, 1,000, 2,000]; ‘max_depth’: [10, 20, 50, 100, 500, None]; ’min_samples_leaf’: [1, 2, 4]; ‘min_samples_split’: [2, 5, 10].

**Bagging meta-estimator (Bagging).** Bagging^47^ classifier is an ensemble method that fits base classifiers each on random subsets of the original datasets and those base classifiers were aggregated to generate a final prediction by voting or averaging. Parameters of RF were searched using grid to maximize AUROC as follows. ‘n_estimators’: [200, 500, 1,000, 2,000].

## 1. 5 Decision Trees

**Decision Trees (DecisionTree).** The Decision Trees^48^ classifier use recursive partitioning to create a tree. Each node in tree represents a cell of the partition. The root node of the tree is spitted into successor children based on a set of splitting rules using features. The splitting is realized in a recursive manner until the subset at a node has all same label, or the maximum depth has been achieved. Parameters of Decision Trees were searched using grid to maximize AUROC as follows. ‘max_depth’: [10, 20, 50, 100, 500, None]; ’min_samples_leaf’: [1, 2, 5, 10]; ‘min_samples_split’: [2, 5, 10, 15, 100].

**Extremely randomized trees (ERT).** Extremely randomized trees^49^ are a new tree-based ensemble methods, which selects its cut-point fully at random, i.e., independently of the target label, for a given attribute or feature. At each tree node, this is combined with a random choice of a certain number of features and the best one among features is determined. Parameters of ERT were searched using grid to maximize AUROC as follows. ‘max_depth’: [10, 20, 50, 100, 500, None]; ’min_samples_leaf’: [1, 2, 5, 10]; ‘min_samples_split’: [2, 5, 10, 15, 100].

## 1.6 Naïve Bayes

The key basis of Naïve Bayes classifier is Bayes theorem and assumes that all the features are independent of each other. Bayes theorem can be described as

$$P\left( A | B \right)=P(A)\frac{P(B|A)}{P(B)}$$

Here $A$ and $B$ are two events and $P\left( A | B \right)$ is the probability of one event when another event has already been occurred. $P(A)$ is the probability of event $A$ which is independent with the proability of event $B$.

**Gaussian Naïve Bayes (GaussianNB).** In Gaussian Naïve Bayes^51^, the likelihood of the features is assumed to be Gaussian distribution

$$P\left( X_{i} | y \right)=\frac{1}{\sqrt{2\pi\sigma_{y}^{2}}}exp(-\frac{{(X_{i}-\mu_{y})}^{2}}{2\sigma_{y}^{2}})$$

The parameters $\sigma_{y}$ and $\mu_{y}$ can be estimated using maximum likelihood. Parameters of GaussianNB were searched using grid to maximize AUROC as follows. ‘var_smoothing’: [1e-9, 1e-10, 1e-11].

**Bernoulli Naïve Bayes (BernoulliNB).** In Bernoulli Naïve Bayes^50^, the likelihood of the features is assumed to be Gaussian distribution

$$P\left( X_{i} | y \right)=P\left( i | y \right)X_{i}+(1-P(i|y))(1-X_{i})$$

The continuous features are binarized using the default threshold 0. Parameters of BernoulliNB were searched using grid to maximize AUROC as follows. ‘alpha: [0.01, 0.1, 0.5, 1.0, 10].

## 1.7 Neural Networks

**Multi-layer Perceptron (MLP).** Multi-layer Perceptron^52^ is a supervised learning model that learns any continuous function to map the input features to target output. MLP usually consists of three layers: input layer, output layer and a hidden layer. Similar to a feed forward network, data in MLP flows in the forward direction from input layer to output layer and MLP is trained with the back propagation learning algorithm. Parameters of MLP were searched using grid to maximize AUROC as follows. ‘hidden_layer_sizes: [(50, 50, 50), (50, 100, 50), (100,)]; ‘activation’: [‘tanh’, ‘relu’]; ‘solver’: [‘sgd’, ‘adam’]; ‘alpha’: [‘0.0001’, ‘0.05’]; ‘learning_rate’: [‘constant’, ‘adaptive’].

**Attentive Interpretable Tabular Learning (Tabnet).** Tabnet^31^ uses deep neural networks for tabular data with inputting raw table data without any preprocessing and is trained using gradient descent-based optimization. It uses sequential attention to choose which features to reason from at each decision step, which enables interpretability and better learning. Tabnet shows superior performance across tabular datasets from different domains. Parameters of MLP were searched using grid to maximize AUROC as follows. ‘n_d’: [8, 10, 16, 32]; ‘n_a’: [24]; ‘gamma’: [0.5, 1, 1.5, 2]; ‘n_independent’: [2].

**Multi-Omics Graph Convolutional Networks (MOGONET).** MOGONET^14^ is a supervised multi-omics integration method for biomedical classification tasks utilizing graph convolutional networks (GCN). MOGONET first constructs a weighted sample similarity network for each type of omics data using cosine similarity. Then, a GCN is trained for each omics data type to generate initial prediction of class labels by using omics features and corresponding similarity network. Those initial predictions are further utilized to construct the cross-omics discover tensor, which is reshaped into a vector and forwarded to View Correlation Discovery Network (VCDN) for final label prediction. We used the default parameters for MOGONET.

# 2. Statistical Analysis

To reduce the dimension of miRNA, mRNA and microbiome omics data, we first filtered out genes whose total count is lower than the total subjects and followed by further filtering with very low counts using the *filterByExpr* function. Then, the data was normalized using the trimmed mean of M values (TMM) method. For *p*-value calculation, we input each gene’s counts-per-million (CPM) values into the *wilcox.test* function in R (v4.0.2). Then, we set a *p*-value cutoff based on an FDR threshold using the Benjamini & Hochberg method. For each three omics data, we selected the top-300 genes/ASVs with the lowest adjusted *p*-value.


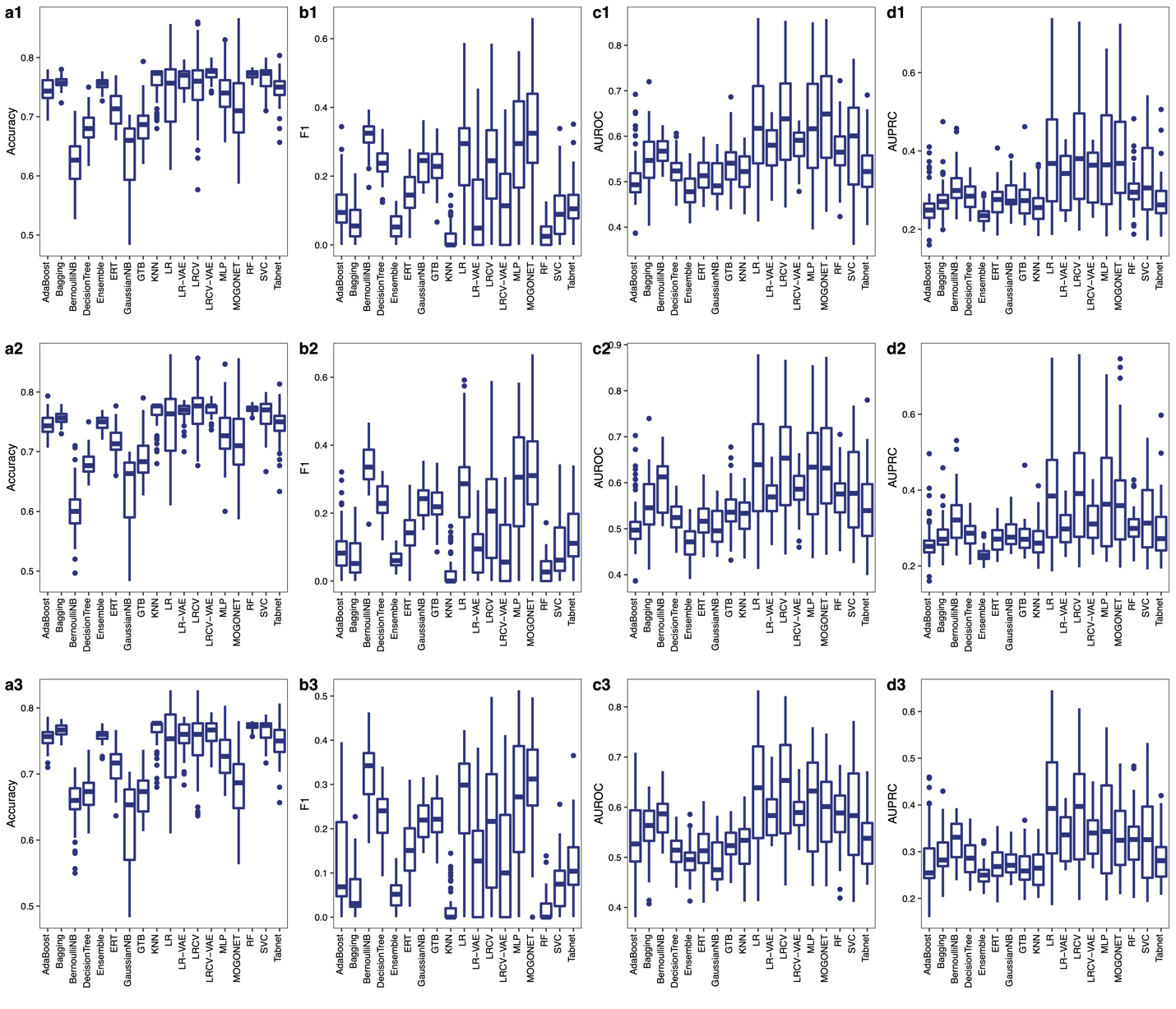


**Figure S1: Prediction performance of each prediction method in cross-validation.** Each classifier was applied to predict the asthma outcome of children at year 3 using all six omics combinations (in total 63). Boxplot shows the performance of each method in all 63 combinations. The missing values of miRNA, mRNA and metabolomics data were imputed using the median (a1-d1); TOBMI (a2-d2) and missForest (a3-d3). Boxes indicate the interquartile range between the first and third quartiles with the central mark inside each box indicating the median. Whiskers extend to the lowest and highest values within 1.5 times the interquartile range.


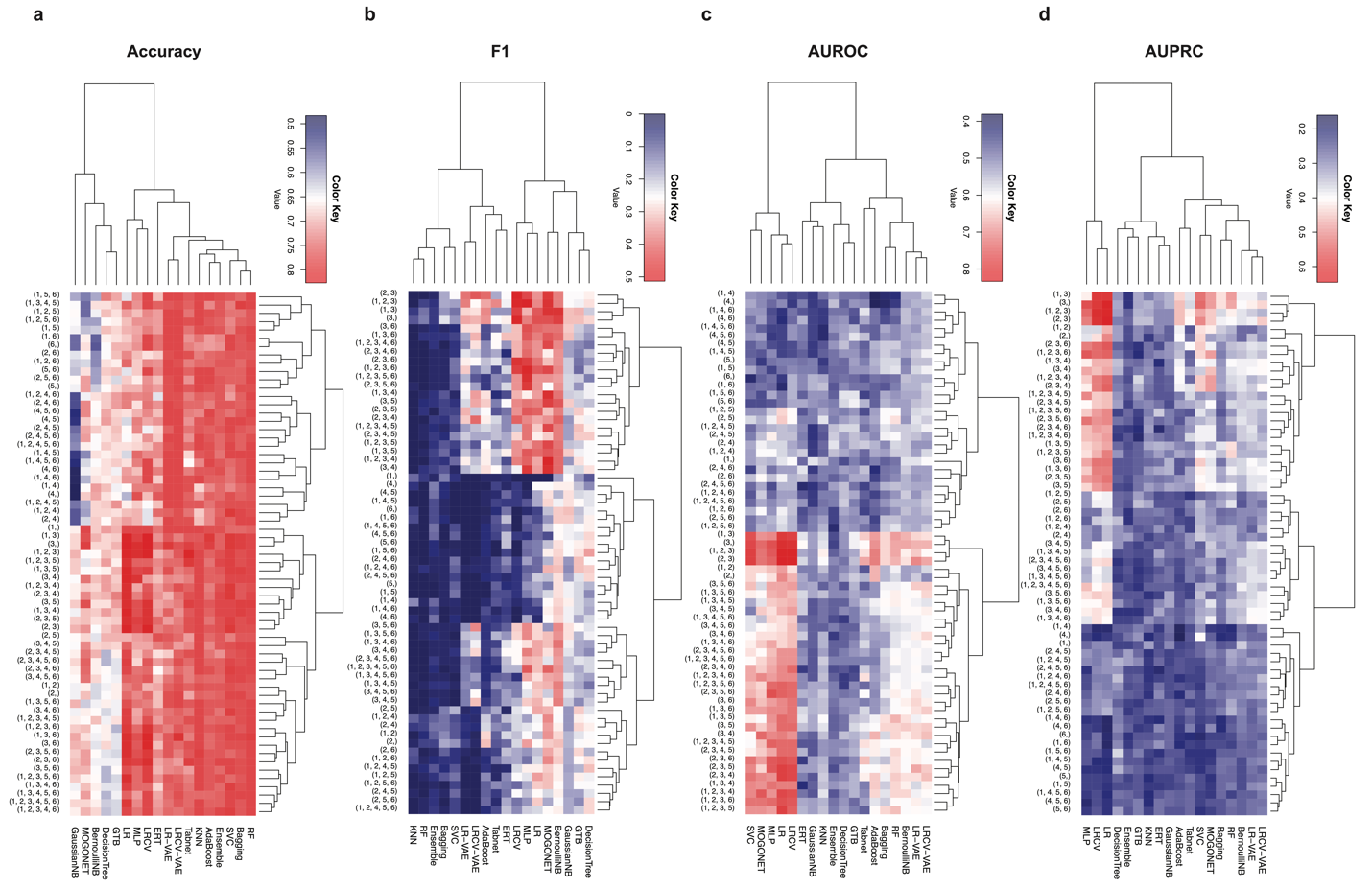


**Figure S2: Prediction performance of classification models using all six omics combinations in cross-validation.** Each classifier was applied to predict the asthma outcome of children at year 3 using all six omics combinations (in total 63). The missing values of miRNA, mRNA and metabolomics data were imputed using the *TOBMI*. 1: GWAS; 2: miRNA; 3: mRNA; 4: Microbiome; 5: Metabolomics; 6: DNA methylation.


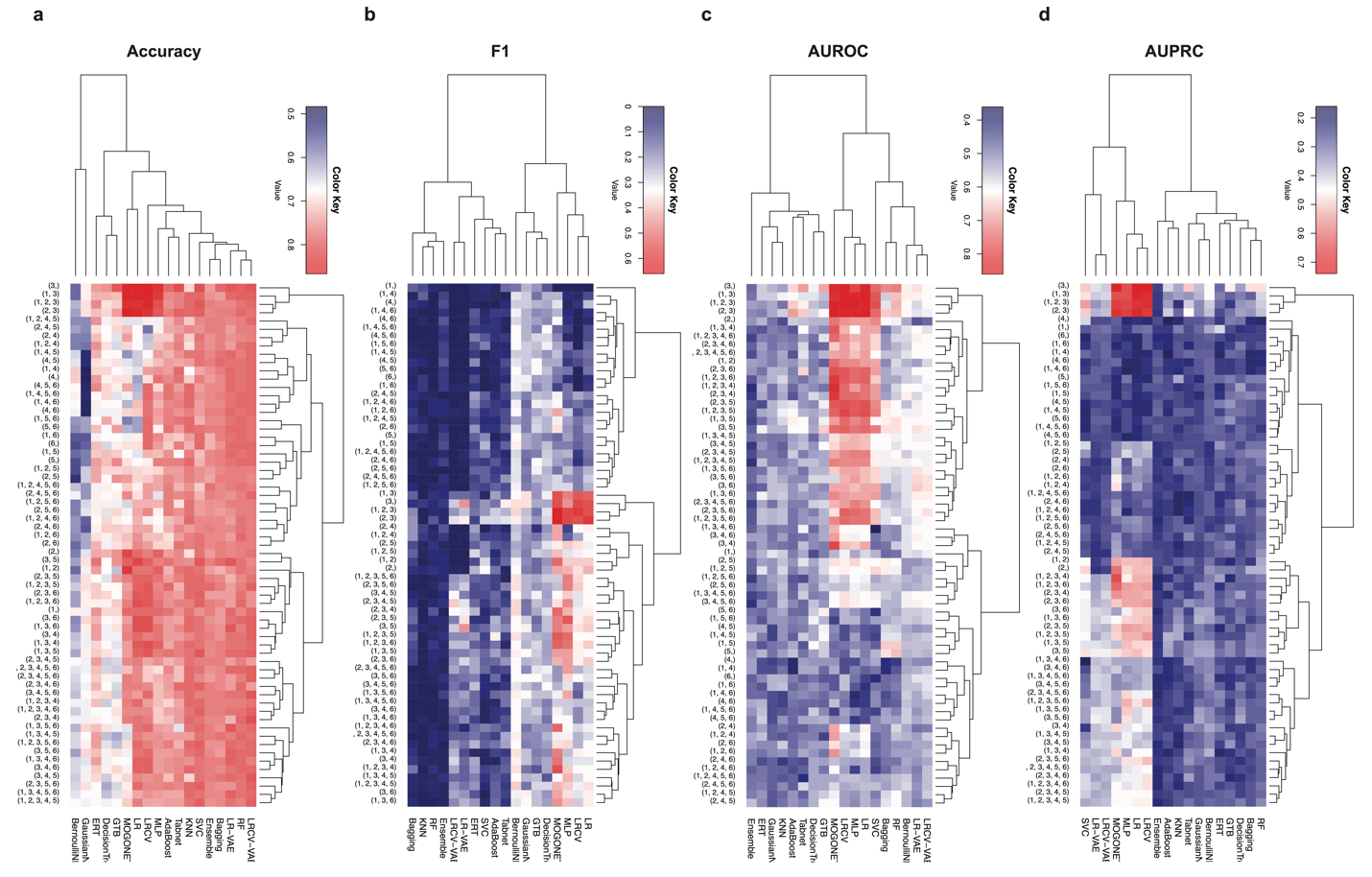


**Figure S3: Prediction performance of classification models using all six omics combinations in cross-validation.** Each classifier is applied to predict the asthma outcome of children at year 3 using all six omics combinations (in total 63). The missing values of miRNA, mRNA and metabolomics data were imputed using the *missForest*. 1: GWAS; 2: miRNA; 3: mRNA; 4: Microbiome; 5: Metabolomics; 6: DNA methylation.


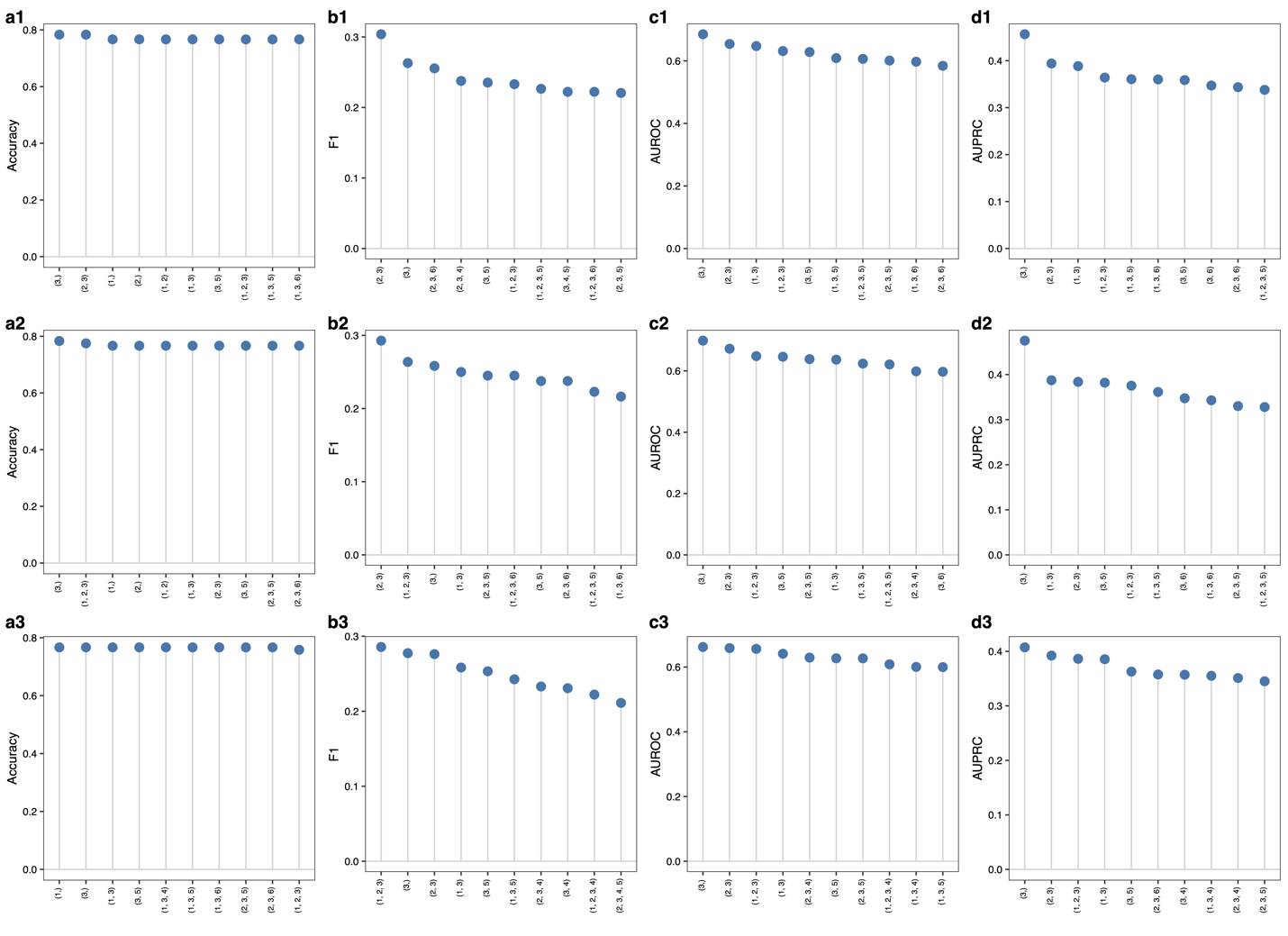


**Figure S4: Omics combination importance in cross-validation.** Each classifier was applied to predict the asthma outcome of children at year 3 using all six omics combinations (in total 63). We show the top-10 most important omics combinations based on the median of prediction performance over 16 methods. The missing values of miRNA, mRNA and metabolomics data were imputed using the median (a1-d1); TOBMI (a2-d2) and missForest (a3-d3). 1: GWAS; 2: miRNA; 3: mRNA; 4: Microbiome; 5: Metabolomics; 6: DNA methylation.

| **Table S1: Important omics biomarkers identified by MOGONET using genome, miRNA and mRNA data.** | |
| --- | --- |
| **Omics data type** | **Biomarkers** |
| mRNA expression (2) | ENSG00000267174, ENSG00000004139 |
| miRNA expression (28) | hsa-miR-581, hsa-miR-376c-3p, hsa-miR-371b-5p, hsa-miR-374b-5p, hsa-miR-374c-5p, hsa-miR-376a-3p, hsa-miR-376a-5p, hsa-miR-376b-3p, hsa-miR-376b-5p, hsa-miR-3691-3p, hsa-miR-376c-5p, hsa-miR-377-3p, hsa-miR-377-5p, hsa-miR-378a-5p, hsa-miR-378f, hsa-miR-378h, hsa-miR-370-3p, hsa-miR-3690, hsa-miR-381-5p, hsa-miR-3680-3p, hsa-miR-323a-3p, hsa-miR-323b-3p, hsa-miR-324-5p, hsa-miR-329-3p, hsa-miR-330-5p, hsa-miR-339-3p, hsa-miR-342-3p, hsa-miR-34a-5p |


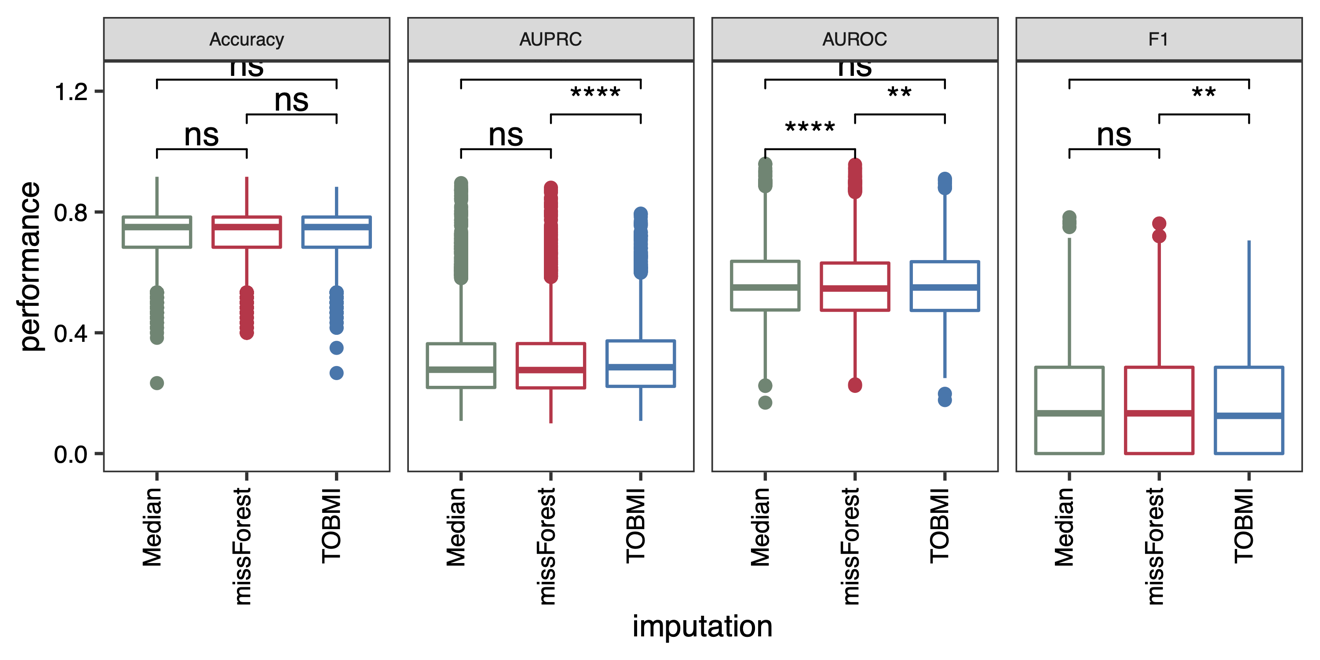


**Figure S5: Performance comparison between different imputation methods.** Boxplot shows the performance of each classification method in each omics combination. P-values were calculated using the paired Wilcoxon test. Boxes indicate the interquartile range between the first and third quartiles with the central mark inside each box indicating the median. Whiskers extend to the lowest and highest values within 1.5 times the interquartile range.


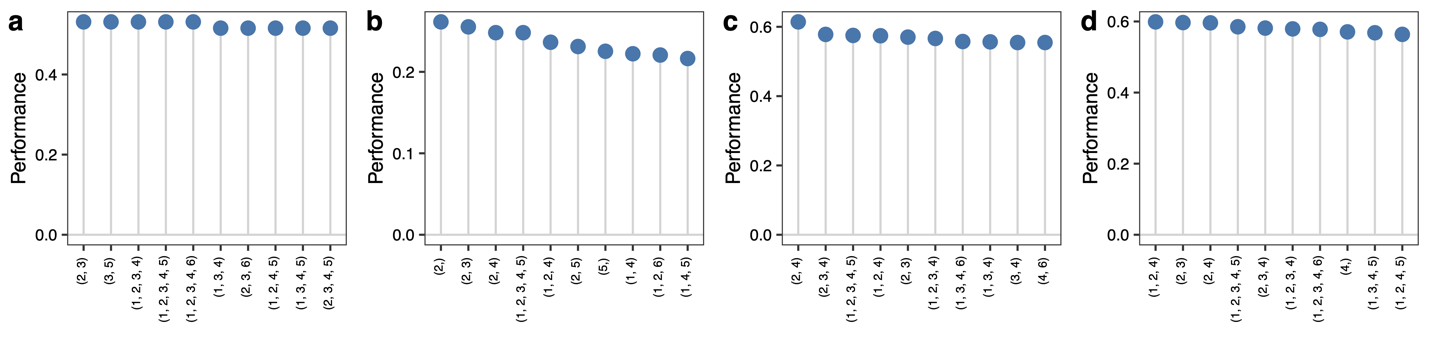


**Figure S6: Omics combination importance in hold-out validation.** Each classifier is applied to predict the asthma outcome of children at year 3 using all six omics combination (in total 63). We show the top-10 most important omics combinations based on the median prediction performance over 16 methods. The missing values of miRNA, mRNA and metabolomics data were imputed using the median values. 1: GWAS; 2: miRNA; 3: mRNA; 4: Microbiome; 5: Metabolomics; 6: DNA methylation.


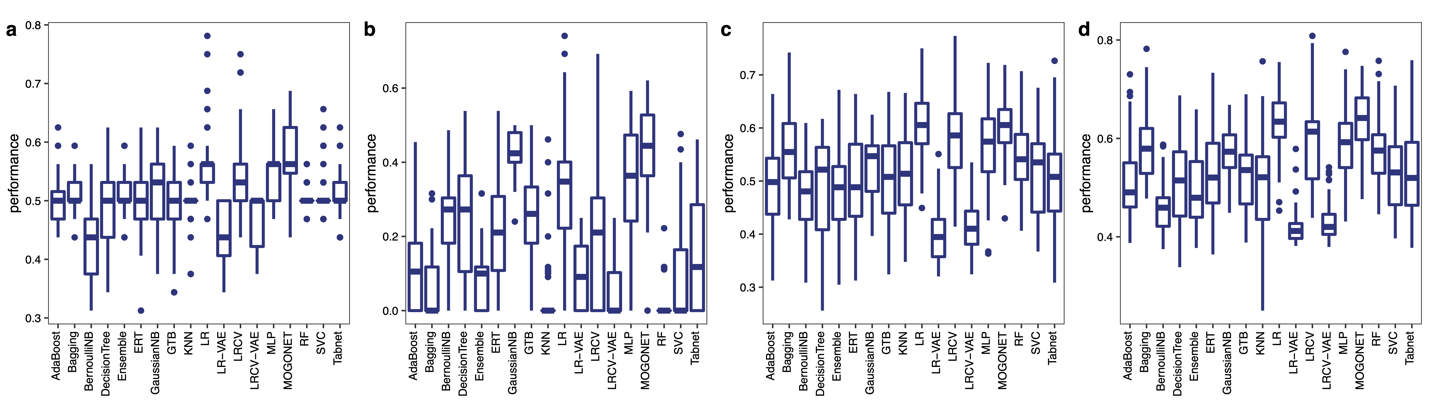


**Figure S7: Prediction performance of each method in hold-out validation.** Each classifier is applied to predict the asthma outcome of children at year 3 using all six omics combinations (in total 63). Boxplot shows the performance of each method in all 63 combinations. The missing values of miRNA, mRNA and metabolomics data were imputed using the median values. Boxes indicate the interquartile range between the first and third quartiles with the central mark inside each box indicating the median. Whiskers extend to the lowest and highest values within 1.5 times the interquartile range.
